# Making the economic case for prevention – a view from Wales

**DOI:** 10.1186/1471-2458-12-460

**Published:** 2012-06-20

**Authors:** Janine Hale, Ceri J Phillips, Tony Jewell

**Affiliations:** 1 Health, Social Services and Children Analytical Team, Welsh Government, 4th Floor, North Core, Cathays Park, Cardiff, CF10 3NQ, UK; 2 Swansea Centre for Health Economics, College of Human and Health Sciences/Swansea University, Singleton Park, Swansea, SA2 8PP, UK; 3 Department for Public Health and Health Professions, Welsh Government, Cathays Park, Cardiff, CF10 3NQ, UK

## Abstract

**Background:**

It is widely acknowledged that adverse lifestyle behaviours in the population now will place an unsustainable burden on health service resources in the future. It has been estimated that the combined cost to the NHS in Wales of overweight and obesity, alcohol and tobacco is in excess of £540 million.

In the current climate of financial austerity, there can be a tendency for the case for prevention efforts to be judged on the basis of their scope for cost savings. This paper was prompted by discussion in Wales about the evidence for the cost savings from prevention and early intervention and a resulting concern that these programmes were thus being evaluated in policy terms using an incorrect metric. Following a review of the literature, this paper contributes to the discussion of the potential role that economics can play in informing decisions in this area.

**Discussion:**

This paper argues that whilst studies of the economic burden of diseases provide information about the magnitude of the problem faced, they should not be used as a means of priority setting. Similarly, studies discussing the likelihood of savings as a result of prevention programmes may be distorting the arguments for public health.

Prevention spend needs to be considered purposefully, resulting in a strategic commitment to spending. The role of economics in this process is to provide evidence demonstrating that information and support can be provided cost effectively to individuals to change their lifestyles thus avoiding lifestyle related morbidity and mortality. There is growing evidence that prevention programmes represent value for money using the currently accepted techniques and decision making metrics such as those advocated by NICE.

**Summary:**

The issue here is not one of arguing that the economic evaluation of prevention and early intervention should be treated differently, although in some instances that may be appropriate, rather it is about making the case for these interventions to be treated and evaluated to the same standard. The difficulty arises when a higher standard of cost saving may be expected from prevention and public health programmes.

The paper concludes that it is of vital importance that during times of budget constraints, as currently faced, the public health budgets are not eroded to fund secondary care budget shortfalls, which are more easily identifiable. To do so would diminish any possibility of reducing the future burden faced by the NHS of lifestyle-related illnesses.

## Background

It is widely acknowledged that adverse lifestyle behaviours in the population now will place an unsustainable burden on health service resources in the future. It is estimated that the ten leading risk factors for disease are responsible for 77% of deaths and 51% of disability adjusted life years (DALYs) in the UK [1]. The main contributors to the DALYs experienced in the UK are tobacco, high blood pressure, high cholesterol, high BMI and alcohol, together accounting for 41% of the burden. It has also been estimated that behavioural causes account for nearly 40% of all deaths in the United States [2]. In contrast it is argued that medical care has a relatively minor role to play in reducing early deaths [3] and that a broad programme of prevention aimed at changing these behaviours may help to reduce this burden [4].

Traditionally, Wales has had an interest in and an emphasis on the economics of public health, that even pre devolution was not reflected in other parts of the United Kingdom. The Heartbeat Wales programme, initiated in 1985 as a national demonstration project for reducing coronary heart disease included economic appraisal [5], while the economics of health promotion were being considered in Wales before the publication of the Wanless reports that highlighted the need to further develop the evidence base in this area [6].

The Review of NHS Funding and Performance, five years after the original Wanless report, stated that the UK has underperformed in terms of potential life years lost for some diseases (ischaemic heart disease, cerebrovascular and respiratory diseases) when compared to other countries [7]. Furthermore, with the continuing rising trend in obesity, results are worse than even the slow uptake scenario, suggesting that significantly higher levels of funding will be required to provide the level of services outlined in the first Wanless review in 2002. The picture is even starker in Wales, with levels of smoking, obesity and alcohol abuse high relative to other countries within the UK.

### The economic case for prevention

Reviews and models that have been carried out suggest that substantial benefits could be realised from systematic prevention efforts [8,9]. For example, it has been estimated that a significant proportion of the past reductions in coronary heart disease mortality could be attributed to preventive efforts, with treatment explaining less of the mortality decline than risk factor changes [10].

In addition to the public health arguments for Government intervention in public health policy, the case can further be made on economic grounds. Economic theory suggests that governments should intervene in the provision and delivery of goods and services when the market fails to provide an adequate supply of them . In the case of prevention, there are at least three potential sources of market failure [8]:

Time inconsistent preferences i.e. the preferences of an individual may not be consistent over time. An individual may choose instant gratification over their long-term interests, such that a commitment made in the present to behave in a certain way in the future will be broken when that time in the future comes;

Departures from rationality (particularly in young people). The assumption that people act rationally (defined as maximising their expected utility) is core to economic thinking. However, it is recognised that children and young people in particular often make choices that may not be in their long-term best interests - they make lifestyle choices with a short-term view, even when they are informed of future consequences. This could provide a justification for government intervention, to prevent them from harming themselves; and

imperfect information – government intervention in the provision and production of health information is justifiable as information itself is a public good and is thus likely to be undersupplied by the market.

The above market failures justify an explicit government role in the prevention of lifestyle-related morbidity and mortality [8].

Further, the existence of negative externalities, whereby some of the costs of the lifestyle behaviour may be borne by other individuals, may also provide justification for government intervention in this area. Negative externalities may arise for example though exposure to second hand smoke or being a victim of accidents or anti social behaviour caused by alcohol misuse of others. Interventions to tackle these can take the form of awareness campaigns to raise knowledge of the impacts of behaviours on others with a view to changing behaviours (for example raising awareness of the impacts of second hand smoke on children), or more direct regulations to control behaviours to reduce the negative externalities, as has been seen with the regulations on smoking in public places.

Making the economic case for prevention, however, has not always been easy. This paper discusses the different types of ‘economic’ arguments that have been put forward and outlines the role that economics can play in making decisions about public health spending. The intention of this paper is to add to discussion surrounding appropriate use of economic arguments to support public health initiatives and is not intended to give a definitive answer. A systematic approach to identifying literature to inform the paper was taken - although a full systematic review was not performed – to capture the range of viewpoints portrayed within the literature, to supplement views obtained as a result of our experience in Wales.

## Discussion

### Costs of lifestyle related illnesses

Whilst cost of illness studies per se are not sufficient information for allocation of resources, the estimation of the economic losses or costs associated with a particular health condition can help to establish the proportion of government resources that could have been used for other purposes in the absence of these, preventable, diseases [11]. Cost of illness studies also need to be treated with a degree of caution as they are often based on estimations and assumptions as the full data set required often does not exist, and there is no standardised methodology for studies of this nature.

#### Obesity

Obesity is a major public health issue with over 500 million people worldwide estimated to be obese and nearly 1.5 billion people overweight [12]. While the total impact is currently unknown [13], estimates have clearly demonstrated that obesity imposes a significant economic burden. For example, it was estimated that in 2002 the direct plus indirect costs of obesity and overweight in England was £6.6 - £7.4 billion [13]. These figures have been projected to increase dramatically, with an estimated additional 11 million more obese adults in the UK by 2030 adding an additional £1.9-£2 billion annual bill to the healthcare system in treatment of preventable diseases [14]. An earlier study predicted that the cost burden of obesity would reach £49.9 billion per annum in England by 2050, as a consequence of increasing BMI [15], and on the basis of these figures, the likely cost impact in Wales by this time would be approaching £3 billion per annum.

Public health action is therefore required to tackle these trends and narrow inequalities in health [16]. Some believe that overweight and obesity will soon rival tobacco as the world’s leading cause of preventable early deaths, with the possibility that the health effects of the obesity pandemic could outweigh the gains in life expectancy achieved through reductions in smoking rates [17].

It is now widely accepted that changing our dietary habits for the better, alongside an improvement in our activity levels, will have a major impact in reducing rates of the chronic diseases [13], thus potentially reducing the economic burden of preventable illness as a result of this lifestyle behaviour. It needs to be recognised however, that to achieve this requires an adequate level of investment, with a long term commitment to a multipronged approach, alongside an acceptance that the payoff on the investment may not be observed for several years [17].

#### Smoking

Overall, it was estimated that smoking caused some 109,164 deaths in the UK in 2005, and the direct cost to the NHS of smoking in the UK was estimated to be £5.2 billion in 2005–6 [18]. Godfrey et al. [19] developed a model using a cohort of smokers, ex-smokers and non smokers to estimate the lifetime health care costs and health consequences of smoking for the population of England. The model estimated that for a cohort of 1000 men, the difference in lifetime direct health care costs between smokers and non-smokers was £8.6 million (£4.1 million when discounted at 3.5%). Furthermore, it has been estimated that the cost of smoking to the NHS in Wales in 2007/08 was £386 million, equivalent to £129 per head and 7 per cent of total healthcare expenditure in Wales [20]. In addition, 34 million working days are lost in England and Wales every year [21].

Whilst there is no agreement on the number of deaths that can be attributed to obesity and physical inactivity combined, it is clear that these and smoking are the top behavioural causes of premature death [2].

#### Alcohol

Alcohol is a major cause of disease and injury, accounting for 9.2% of the years of life lost or lived with a disability [22]. It has been estimated that alcohol use accounts directly for 8,000 potential life-years lost in Wales and can indirectly be accountable for an additional 5,000 [22]. The annual cost of alcohol harm to the NHS in England has been estimated as £2.7 billion [23]. The cost to the NHS in Wales has been estimated as being between £70 million and £73million [24].

Scarborough et al. [25] estimated the costs to the NHS in the UK of poor diet, physical inactivity, smoking, alcohol and overweight and obesity. They found that poor diet has the highest impact on budget of the NHS. Table 1 shows the estimated costs of overweight and obesity, tobacco and alcohol to the NHS in Wales, along with the prevalence of these lifestyles. Avoidable lifestyle related illnesses clearly place major demands on NHS resources, but it must be remembered that there are also implications for the quality and length of life of the Welsh population. Working to change these behaviours therefore brings benefits not only in terms of potentially releasing resources that can be used elsewhere in the NHS, but can also bring improvements to the length and quality of the lives of Welsh citizens.

**Table 1:** Estimated costs of lifestyle related illnesses and prevalence of those lifestyles in Wales

**Risk factor**	**Estimated cost to the NHS in Wales (million)**	**Prevalence in Wales**
**Overweight and obese**^**b**^	£86^a^	57%
Obese^b^		21%
**Tobacco**	£386^c^	
Adult smokers^b^		24%
Adult non-smokers reporting regularly being exposed to other people’s tobacco smoke^b^		33%
Secondary school pupils reporting smoking at least once a week^d^		6%
**Alcohol**	£69.9 - £73.3^a^	
Adults reported drinking above recommended guidelines in past week^b^		45%
Secondary school pupils report drinking at least one alcoholic drink weekly^d^		16%
Secondary school pupils reporting having been drunk 4 or more times in their life^d^		12%

^a^[24].

^b^[26].

^c^[20].

^d^[27].

### Cost effectiveness versus cost saving

While studies of the economic burden of diseases can provide information about the magnitude of the problem faced, they should not be used as a means of priority setting [28,29]. In order to set priorities for the allocation of resources, information is also required on many factors, including the relative cost effectiveness of different intervention strategies [11]. It is also important to avoid the trap of using such estimates to assume that prevention strategies will necessarily lead to cost savings, since successful prevention programmes also require resources.

It is well-recognised that the evidence base for public health is not as well developed, particularly with regards to economic evaluation [4,8]. There is, however, evidence supporting the effectiveness of interventions, even though the cost effectiveness assessment may not have been made. Recent work undertaken for the Department of Health in England outlines that there is effectiveness evidence for interventions aimed at reducing many of the lifestyle risk factors, although it may be more difficult to be conclusive about cost effectiveness in some areas [30]. It is important to note however, that the unavailability of strong evidence of effectiveness does not indicate lack of effectiveness. Suhrcke et al. [8] have argued that Government has an important role to play not only in interventions to prevent lifestyle risk behaviours, but also in research to further develop the evidence base about what works. In a ranking of preventative health interventions produced for Health England, the top 12 all demonstrated cost per QALY figures below £10,000, and well within the recognised threshold of cost-effectiveness, with 8 of these potentially being cost saving [31].

Rappange et al. [32] make the point that prevention has sometimes been promoted as simultaneously improving public health and saving money, but that this is unlikely to be the case, since any savings made in the treatment of the preventable disease are likely to be offset by the treatment costs of an unrelated illness in later life. The authors suggest that the solution is to include the treatment cost of those diseases in later life in the economic assessment. However, this is not employed within health technology assessments of ‘curative’ interventions and to do so only in the assessment of preventive interventions would be introducing an adverse bias for such interventions in determining resource allocation.

There are also other potential issues with this approach. Cost effectiveness assessments are usually about assessing the technical efficiency of a programme, assessing in terms of achieving a single, narrow objective. To include wider impacts would need cost consequences or cost benefit analyses to be carried out. If the perspective is going to be widened to include the costs of treating other illnesses, what about wider impacts on benefits - do we adjust the QALYs gained by the preventive intervention to reflect the possibility of these illnesses in later life? Furthermore, there may be additional benefits in terms of reductions in risks of other diseases in addition to the target disease as a result of a successful public health programme, while there may also be social diffusion effects. Calculating and including the cost of future unrelated illnesses assumes that the treatments themselves and the costs associated with them remain constant over time. Given that these may occur well into the future, how realistic an assumption is this? For example, it has been argued that “reductions in premature deaths [from reductions in smoking prevalence] will not necessarily result in increased health care costs in the future. Improvements in lifestyle achieved through reduced smoking may, however, reduce disease and disability in the elderly. This will place fewer demands on the health services and release resources for other health needs. In addition, a reduction in premature deaths will provide benefits to the NHS through reductions in the loss of NHS employees and their associated skills and experience” [5]. This is clearly an area that needs further discussion in the context of all economic evaluations that relate to health outcomes.

Barton et al. [9] have used modelling techniques to demonstrate that a population programme that reduces cardiovascular events by just 1% could result in significant savings for the NHS. This study follows the currently accepted techniques of economic appraisal and thus does not capture the costs of possible future illnesses as proposed by Rappange et al. Studies of this nature provide important indications of the potential for saving costs from avoidable morbidity and mortality, and also demonstrate the scale of resources that could be allocated to these preventative programmes before reaching the likely threshold of cost effectiveness. Even when the savings that are generated may be outweighed by the costs of future possible illnesses, it is still worth preventing (and thus saving expenditure on) avoidable illnesses to improve the efficiency of the NHS overall. An important distinction needs to be drawn between possible savings within a particular disease area and reducing the overall NHS budget. The former are important and worth pursuing even if the overall effect on the latter is difficult to prove.

Whilst some of the issues above can be dealt with through the adoption of a social welfare perspective for the evaluation of public health programmes as suggested by Weatherly and colleagues [33], this doesn’t entirely address the point. If different economic evaluations adopt different perspectives for analysis, how can comparisons be made across programmes?

The issue here is not one of arguing that the economic evaluation of prevention and early intervention should be undertaken differently, although in some instances that may be appropriate, rather it is about making the case for a consistent approach to be employed across all evaluations. It needs to be recognised however, that the application of economic evaluation techniques developed and increasingly recognised as the standard approach for health care interventions [NICE reference case: http://www.nice.org.uk/media/B52/A7/TAMethodsGuideUpdatedJune2008.pdf, to public health programmes is not necessarily straightforward [6], and these potential difficulties need to be given careful consideration in the interpretation of results. Notwithstanding these issues, a further difficulty arises when prevention and public health programmes are viewed as potential sources of financial savings for the NHS.

### Spend on prevention

Butterfield et al. [34] estimated that in 2006/07, the share of total health expenditure allocated to prevention and public health in England was just over 5 per cent at £5 billion, falling to 4 per cent (£3.7 billion) if spend on pharmaceuticals was excluded.

In Wales, Phillips et al. [35] developed an estimate of prevention spend with reference to the OECD System of Health Accounts and the structure used to develop the estimate produced for England by Health England. This estimate was based on a number of assumptions that received a limited amount of validation by “experts”, but result in an estimate that needs to be used with caution and particularly if using it for comparisons between countries and over time. The estimate suggested that in 2008/09 £280 million was spent on prevention and public health in Wales, which equates to £94 per head, or 5.23 per cent of the Department for Health and Social Services budget, falling to 4.11 per cent if spending on pharmaceuticals was excluded [35].

The conclusion of the report above stated that the system of accounts needs to be amended whereby the basic framework of the OECD Health Accounts is used as the basis of a system applicable to Wales. Without this change, establishing an estimate suitable for comparative purposes between countries and across time would be very difficult.

The need for such accurate and timely health information becomes increasingly important when faced with the marketing efforts of industries selling products that can be detrimental to health. It has been estimated for example, that for every $1 spent by the World Health Organisation on trying to improve the nutrition of the world’s population, $500 is spent by the food industry advertising processed foods [36]. The total advertising spend in the UK on all types of food, soft drinks and chain restaurants was estimated to be £727 million in 2003, with 72 per cent (£522 million) of that being spent on television advertising [37]. Tables 2,3 and 4 show the breakdown of the television advertising spend, alongside household final consumption expenditure. Of additional concern, advertising on food, chain restaurants and soft drinks constitutes almost one third of the advertising seen by children during children’s airtime [38]. Furthermore, it has been estimated that the UK alcohol industry spends approximately £800 million per annum promoting its products, with other sectors of business also playing a significant part in creating a pro-alcohol environment [39]. Hastings and Angus also discuss the detrimental impact of this advertising on children and young people in particular.

**Table 2:** Spend on prevention

	**Spend on Prevention (£ million)**
England^a^	5,000
Wales^b^	280
England and Wales	5,280

^a^[34].

^b^[35].

**Table 3:** UK Advertising expenditure

**Category**	**UK Advertising Expenditure (£ million)**
TV advertising – soft drinks^c^	63
TV advertising – chain restaurants^c^	70
TV advertising - confectionery^c^	107
TV advertising – prepared/convenience foods^c^	128
Advertising spend by UK alcohol industry^d^	800

^c^[38].

^d^[39].

**Table 4:** Household final consumption expenditure

**Category**	**Household Final Consumption Expenditure (£ million, 2009 figure)**^**e**^
**Mineral water and soft drinks**	7,356
**Restaurants and hotels -catering**	75,726
**Sugar and sweet products**	8,666
**Alcoholic beverages**	
Retail	14,890
Restaurants and hotels	26,217
**Tobacco**	16,356

^e^[40].

Whilst the figures in Tables 3 and 4 may not seem that large in comparison to the spend on prevention (Table 2), this is only a small proportion of the money spent promoting these products. Sponsorship has not been included above and consideration needs to be given to the mixed messages that are sent when major sporting and other high profile events are sponsored by fast food and confectionary producers. Named sponsors for the Olympics to be held in London 2012 include for example, McDonalds, Cadbury and Coca Cola, with McDonalds being named as the only branded restaurant at the Olympics venues, and announcing plans to open the world’s largest McDonalds restaurant in the Olympic Park [41]. Whilst it has been argued that other food will be available in unbranded venues, offering choice and balance, it is the branding that will be seen and the messages that this sends that needs careful consideration when decisions such as this are taken.

### The balance between prevention and treatment

In the United States only 2 to 5 percent of the total health budget is allocated to population-wide approaches to health improvement, despite behavioural choices being estimated as explaining 40% of premature death [42] and approximately 70% of the burden of illness and associated costs [43]. In Germany, seven preventable behavioural risk factors have been suggested as accounting for 60% of Germany’s mortality [8]. It has been argued however, that despite a shift in many countries towards a more public health oriented strategy, there is evidence that even the most attentive countries would be well served to put a significantly larger proportion of their health care budgets into public health efforts [3]. Liu and O’Dougherty [42] suggest that a government can improve its allocation of resources at the macro level for financing public health services by regulating the proportion of the health budget that should be allocated to public health and setting up a budget that is separated from the overall health budget, although they make no recommendations as to what the proportion should be.

Improving population health could enhance the productivity of the workforce and boost the national economy, reduce health care expenditures and most importantly, improve people’s lives [2]. There is, for example, increasing evidence of the beneficial health and economic effects of comprehensive smoke-free public places legislation [44-48]. It needs to be borne in mind however, that prevention does not always save money, but this should not detract from the need to rebalance health care systems towards disease prevention and health improvement [3]. The investment in better health in itself is of value. The rationale behind increasing expenditure on public health is the intrinsic value placed on the health it confers on the population, not its monetary savings [3]. Suhrcke et al. [8] argue that prevention should not be held to a higher standard than medical care, where cost-saving is not the prime objective. Liu [49] argues that although there is evidence that allocation of more resources to primary and preventive care would improve allocative efficiency, there is no clear mechanism for achieving the desirable resource shifts.

Daube [50] suggests that public health is the poor relation in the health system. He suggests that a modest increase in the allocation to prevention would enable significant advances to be made across a wide range of public health activity and research areas. It is widely recognised however, that public health measures are inadequately funded, thus undermining their effectiveness [3]. In a study examining what incentives there are for NHS managers to look at wider health issues, Hunter and Marks [51] found that public health resources and staff are thinly spread and that public health leadership was missing. Furthermore, this study found that although there was an apparent shift in the rhetoric towards prevention, the incentives in the system were still all geared towards secondary care, with managers and indeed governments being preoccupied by the demands of the acute care sector.

Public health practitioners need to be able to influence the budget for public health activities in order that the longer term issues are not omitted in favour of short-term demands. Maher and Ford [52] argue that especially in times of economic crises, policy makers and decision makers need effective persuasion when deciding the allocation of resources, and that the case for investment in disease prevention and management should be made in terms of promoting health as a human right and a mechanism for contributing towards poverty reduction and economic stability. This is crucial as public health resource needs are always in competition with the needs of clinical services. The latter nearly always take precedence – treatment of individual patients seems far more immediate a priority than changes in health status for the future [53].

Some authors suggest that society may value health gains from preventive interventions differently to those achieved from other health interventions, in part due to the inability to directly identify those that benefit, but also due to issues of personal responsibility, and that this may impact on willingness to fund the programmes [32,54]. This could be argued to strengthen the case for Government provision of these services, as in the absence of this provision, these programmes may not be given any priority at all. Given this however, the appropriate level of funding for public health is subject to political considerations as those requiring clinical services are often immediately identifiable, whilst those that will benefit from improved future health status are not. Coote [55] suggests that a rebalancing of investment towards prevention is unavoidable, but that the pace of change will need to be carefully managed to ensure that there is no public perception that the quality of health services is in decline as a result. It is clear therefore, that the economic case for any public health programme is only one component of the decision making process and that these other factors also need to be considered.

The inability to reliably identify expenditure on public health potentially increases the ease with which public health budgets can be reallocated when budgets are being reduced, as no systematic monitoring is taking place [53].

Our Healthy Future [56] renews the Welsh Government’s commitment to improve the quality and length of life and to ensure that everyone in Wales has a fair chance to lead a healthy life. The aims of Our Healthy Future include increasing the pace of change in improving health in Wales and providing the strategic direction for national and local public health. Within the framework, there are two actions that are particularly pertinent to this paper:

· Rebalance health and social services to prevention and early intervention;

· Review spend on prevention and early intervention.

The review of spend will help to address some of the concerns outlined above, by establishing a definition of prevention and early intervention that can be tracked and monitored over time. The main purpose of the review of spend is seen as supporting the rebalancing of health and social services to prevention and early intervention. A strategic approach to prevention spend is however still required. The budget for health as a whole and how that is allocated across different categories needs to be examined, rather than the case for prevention being made on a programme by programme basis. Prevention needs to be seen as the responsibility of the whole health system, not just public health practitioners.

Economic approaches could also make an important contribution to the work being undertaken to rebalance health and social services. Programme budgeting and marginal analysis techniques could help to establish explicit mechanisms by which transfers could be made between health and social services and prevention and early intervention.

Research has been undertaken to explore the use of economic evaluation techniques in public health, and preliminary results suggest that cost benefit analysis and cost utility analysis are the preferred approaches to use for informing prioritization decisions. The research also demonstrated that further information was also desirable, for example benchmarks to place net-benefit estimates from cost-benefit analyses into context [57] while the challenges and barriers to wider adoption of economic evaluation techniques in public health require further consideration and the development of a broader framework for undertaking economic evaluations in prevention and public health per se.

### Summary

Whilst burden of disease studies make an important contribution in establishing the size of the problem being faced, these are only part of the picture and care needs to be taken to ensure that they don’t receive more focus than merited. Similarly, discussions around the potential of prevention to save resources are also interesting and can be helpful in informing planning, but the case for prevention should not be made on the basis of these and these studies should not divert attention away from the key question that needs to be addressed, that of the most efficient way of improving population health.

Prevention spend needs to be considered purposefully, resulting in a strategic commitment to spending. Studies demonstrating the size of the problems to be tackled can help inform this and although this paper has shown that these are not insignificant, these are not and should not be the main driver. The existence of market failures is also important and further strengthens the case for a strategic commitment to prevention efforts. The role of economics in this process is to provide evidence demonstrating that information and support can be provided cost effectively to individuals to change their lifestyles thus avoiding lifestyle related morbidity and mortality. There is growing evidence that prevention programmes represent value for money using the currently accepted techniques and decision making metrics such as those advocated by NICE. Where the evidence of effectiveness and cost effectiveness exists, the case should be made to provide that information and support to help individuals live longer, happier, healthier lives, irrespective of whether there are long term cost savings for the NHS as a result.

In an era of limited resources, the challenge in purchasing population health is to find the optimal balance of resource allocation across the known determinants of health that will produce the most maintenance or improvement for the most people with the resources available [58]. It is of vital importance that during times of budget constraints, as currently faced, the public health budgets are not eroded to fund secondary care budget shortfalls, which are more easily identifiable. To do so would diminish any possibility of reducing the future burden faced by the NHS of lifestyle-related illnesses.

## Abbreviations

NHS, National Health Service; NICE, National Institute for Health and Clinical Excellence; BMI, Body Mass Index; QALY, Quality Adjusted Life Year; OECD, Organisation for Economic Co-operation and Development.

## Competing interests

The authors declare that they have no competing interests.

## Authors’ contributions

All authors participated in the design of the study. JH drafted the manuscript. CP and TJ commented on revisions to the manuscript. All authors read and approved the final manuscript.

## Authors’ information

Janine Hale is a Principal Research Officer (Health Economics) at the Welsh Government and also a Honorary Research Fellow with the Swansea Centre for Health Economics and Swansea University.

Ceri Phillips is Professor of Health Economics at Swansea University and Director of the Swansea Centre for Health Economics.

Tony Jewell is the Chief Medical Officer for Wales.

## Pre-publication history

The pre-publication history for this paper can be accessed here:

http://www.biomedcentral.com/1471-2458/12/460/prepub
